# Interplay of *ST2* downregulation and inflammatory dysregulation in hypertrophic cardiomyopathy pathogenesis

**DOI:** 10.3389/fcvm.2025.1511415

**Published:** 2025-06-04

**Authors:** Xingyu Cao, Huawei Wang, Zunsong Hu, Wenfang Ma, Peng Ding, Huang Sun, Xiying Guo

**Affiliations:** ^1^ Department of Cardiology, The First Affiliated Hospital of Kunming Medical University, Kunming, Yunnan, China; ^2^ Department of Computational and Quantitative Medicine, Beckman Research Institute of City of Hope, Duarte, CA, United States; ^3^ Department of Systems Biology, Beckman Research Institute of City of Hope, Duarte, CA, United States

**Keywords:** hypertrophic cardiomyopathy (HCM), *ST2*, inflammation, myocardial fibrosis, RNA sequencing

## Abstract

**Background:**

Hypertrophic Cardiomyopathy (HCM) is an inherited heart disease and the pathogenesis of HCM involves genetic mutations, hemodynamic stress, and metabolic factors, with myocardial fibrosis playing a crucial role in severe clinical events. IL-33/ST2 signaling pathway known for its roles in immune response and tissue repair, participates in cardiac protection and anti-cardiac fibrosis in heart failure. The role of *ST2* in HCM remains unclear, and IL-33/ST2 pathway and broader inflammatory responses may be critical in HCM.

**Methods:**

We re-analyzed RNA sequencing data from 9 high-throughput sequencing datasets comprising myocardial tissue samples from 109 HCM patients and 210 non-HCM controls. Differential gene expression analysis, correlation analyses, and Gene Set Enrichment Analysis (GSEA) were employed to explore the biological significance of *ST2*-related genes and the IL-33/ST2 pathway. Immune infiltration was assessed using CIBERSORTx, and protein-protein interaction networks were constructed using the STRING database.

**Results:**

Our analysis identified 2,660 upregulated and 403 downregulated genes for HCM in the combined dataset, with significant downregulation of the *ST2* gene (log2 fold change = −5.0, adjusted *P*-value = 9.2  ×  10^−^¹⁴³). This downregulation was consistently observed across multiple individual studies. Correlation analysis revealed significant positive correlations between *ST2* and key inflammatory mediators such as *IL6* and *CD163*. GSEA highlighted the enrichment of pathways related to immune response, inflammation, and cardiac morphogenesis, with notable upregulation of pro-inflammatory pathways. Immune infiltration analysis revealed a significant inverse correlation between *ST2* expression and regulatory T cells (*r* = −0.34) and a positive correlation with neutrophils (*r* = 0.39). Pathway analysis indicated *ST2*'s key role in networks involving inflammatory and fibrotic responses.

**Conclusions:**

Our findings suggest that downregulation of *ST2* in HCM may be associated with a dysregulated inflammatory gene network, potentially contributing to myocardial fibrosis and remodeling. These results highlight the possible critical role of the IL-33/ST2 pathway in disease progression, offering a potential therapeutic target for managing inflammation and fibrosis in HCM.

## Introduction

Hypertrophic Cardiomyopathy (HCM) is a leading cause of sudden cardiac death and the most common genetic heart disease (1). Initially considered an autosomal dominant inheritance, various sarcomere genes such as *MYH7, MYBPC3, TPM1, TNNT2* and *TNNI3*, have been identified as key contributors to HCM pathogenesis (2). The efficacy of detecting pathogenic or likely pathogenic variants significantly depends on the sequencing technique. Gene panel sequencing, which focuses on sarcomere genes, typically detects about 46%–55% of these variants (3). While broader whole-exome or genome sequencing identifies potential pathogenic variants in 42%-87.5% of cases, depending on the study (4, 5). Large-scale cohort studies suggest that the overall positivity rate of genetic findings in HCM ranges from 30%–60% (6, 7), highlighting the genetic complexity of the disease. Nevertheless, a significant number of HCM patients remain without identifiable genetic mutations, suggesting that non-genetic factors such as epigenetic, hemodynamic, and metabolic factors might also play crucial roles in the disease's phenotypic expression. Moreover, beyond myocyte hypertrophy and disarray, the presence of myocardial fibrosis—ranging from microscopic levels to significant macroscopic scarring—plays a critical role in the progression of HCM (8). Evidence suggests that severe clinical events, including sudden death, are often more prevalent during pathological transitions and phases of remodeling phases (9, 10).

Although HCM has traditionally been considered a non-inflammatory disease, recent research underscores the intricate involvement of inflammatory processes in the pathological changes associated with HCM. Emerging evidence highlights the significant role of inflammation in myocardial remodeling, with cardiomyocytes and cardiac fibroblasts producing inflammatory cytokines such as TNF-α, IL-6, and IL-1β, which exacerbate myocardial hypertrophy, fibrosis, and ventricular stiffness, ultimately influencing the clinical course of HCM (11–13). Identifying the divergence points in cardiac-specific hypertrophic, inflammatory, and fibrotic pathways is crucial for understanding the disease's distinct phenotypes (14).

Interleukin (IL)-33 (IL-33), a cytokine in the IL-1 family, is known for activating both innate and adaptive immunity (15). Released as an alarmin from damaged cells following tissue injury, IL-33 plays a pivotal role in immune cell activation and tissue repair and immune cell activation (16). The *IL1RL1* gene (also known as *ST2, DER4*, *T1*, and *FIT-1*; throughout this paper, we refer to it as *ST2* for clarity) is located on human chromosome 2q12 and encodes a receptor in the interleukin-1 receptor family. Specifically, its membrane-bound form, known as ST2 Ligand or ST2 Long form (ST2l), plays a critical role in the IL-33/ST2 signaling pathway, leading to the transcription of inflammatory genes and the induction of immune responses (17). This signaling pathway is recognized for its role in cardiac protection, with IL-33 produced by fibroblasts mitigating the maladaptive pro-hypertrophic and pro-fibrotic responses of the myocardium to biomechanical stress (18). The *ST2* gene also encodes soluble ST2 (sST2), a truncated form of the receptor that acts as a decoy, silencing IL-33/ST2l signaling and promoting pro-fibrotic and pro-apoptotic pathways. sST2 has emerged as a promising biomarker for diagnosing and prognosticating heart failure, acute myocardial infarction, and myocarditis, given its stability and lack of influence from factors such as age, atrial fibrillation, or renal function (19).

However, the role of *ST2* in HCM remains unclear. Limited clinical research indicates that sST2 levels may increase in HCM patients (20, 21) and correlate with left ventricular wall thickness, NYHA functional class, and non-sustained ventricular tachycardia (21), suggesting that the *ST2* gene may play a significant role in HCM. Additionally, initial data from RNA sequencing analyses indicate significant regulatory changes in inflammation-related genes in HCM and cardiac fibrosis (22, 23). These findings suggest a potential interplay between the IL-33/ST2 pathway and broader inflammatory responses in HCM, which may play a significant role in the pathophysiology of the disease.

Hence, this study aims to explore the hypothesis that the downregulation of the *ST2* gene and the dysregulation of inflammatory pathways represent a novel mechanism in HCM. Understanding these mechanisms could provide deeper insights into HCM and pave the way for new therapeutic strategies targeting inflammation-related pathways to mitigate disease progression in HCM patients.

## Methods

### Data acquisition

In this study, we re-analyzed raw whole-transcriptome sequencing data using a standardized bioinformatics pipeline. Data were obtained from the Gene Expression Omnibus (GEO) database following a comprehensive search aimed at minimizing selection bias. To ensure data quality and comparability across studies, only datasets that met the following criteria were included: (1) RNA sequencing data generated using paired-end sequencing protocols; (2) availability of complete metadata for each sample; and (3) confirmation that all tissue samples were derived from human heart tissue. Nine high-throughput sequencing datasets fulfilled these inclusion criteria and were selected for analysis: GSE133054, GSE130036, GSE141910, GSE106997, GSE89714, GSE145154, GSE180313, GSE188238, and GSE206978. HCM cases were included regardless of subtype or genotype, provided that the diagnosis was clinically confirmed. Control samples were selected based on the absence of major cardiovascular conditions, including heart failure, coronary artery disease, myocarditis, or documented arrhythmias such as tachycardia. A total of 109 samples from patients with HCM and 210 normal control samples were included in this study.

### Data processing and normalization

The raw sequencing reads were aligned to the human reference genome (GRCh38) using the STAR package (v2.7.6a). Gene annotation downloaded from the Ensembl database (v102) was used for STAR mapping and the following read count evaluation. Then the Picard (v2.26.11) was used to mark duplicates and generate the final bam files. HTSeq-count was employed to quantify the read counts for each gene, resulting in a count matrix. Then gene expression level was normalized by the variance stabilizing transformation (VST) algorithm in the DESeq2 package. The ComBat function in the sva R package was used to correct the batch effects introduced by different sources of studies. With the VST gene expression data, R package umap was used to generate the Uniform Manifold Approximation and Projection (UMAP) plots using the top variable genes (based on median 2 absolute deviation).

### Differential expression analysis

Differential expression analyses were conducted using DESeq2. Differentially expressed genes (DEGs) were identified based on an absolute log2 fold change (log2FC) >1.5 and an adjusted *P*-value (Benjamini-Hochberg method) <0.05. The results were visualized using volcano plots to highlight the most significantly altered interested genes.

### Correlation analysis

Spearman correlation analysis was performed to examine the relationship between *ST2* expression and the expression levels of all other protein-coding genes. Correlation coefficients (r) and associated *P*-values were calculated to identify genes significantly correlated with *ST2*. Heatmap was generated by pheatmap R package based on the features showing high correlation with *ST2* expression.

### Gene set enrichment analysis (GSEA)

Gene Set Enrichment Analysis (GSEA) was conducted using the GSEA software (https://www.gsea-msigdb.org/gsea/doc/GSEAUserGuideFrame.html) to explore the biological significance of genes correlated with *ST2*. Gene sets were derived from the Kyoto Encyclopedia of Genes and Genomes (KEGG) pathways and Gene Ontology (GO) terms. The ranked correlation coefficient of each gene with *ST2* was used as input to GSEA, and enrichment scores were calculated to identify significantly enriched pathways. Only pathways with a false discovery rate (FDR) <0.1 were considered statistically significant.

### Immune infiltration analyses

To estimate the immune cell composition in myocardial tissue samples, we utilized CIBERSORTx, a computational tool that deconvolutes gene expression data to infer the proportions of various immune cell types. The LM22 signature matrix, which includes 22 immune cell phenotypes, was applied to our transcriptomic data. The immune cell fractions were compared between high and low *ST2* expression levels in HCM samples. *ST2* expression was categorized based on the median value of normalized expression within the dataset. Consequently, samples exhibiting *ST2* expression above this median were classified as high expression, whereas those below were classified as low expression. The Wilcoxon test was used to assess the statistical significance of the differences in cell type composition between these groups.

### Network analysis

Network analyses were performed to elucidate the functional interactions and regulatory networks involving *ST2*. Protein-protein interaction (PPI) networks were constructed using the STRING database, focusing on genes significantly correlated with *ST2*. Functional modules and hub genes within the network were identified using Cytoscape software, and pathway enrichment analysis was conducted to highlight key biological processes.

## Results

### Differential expression analysis

**Figure 1:**
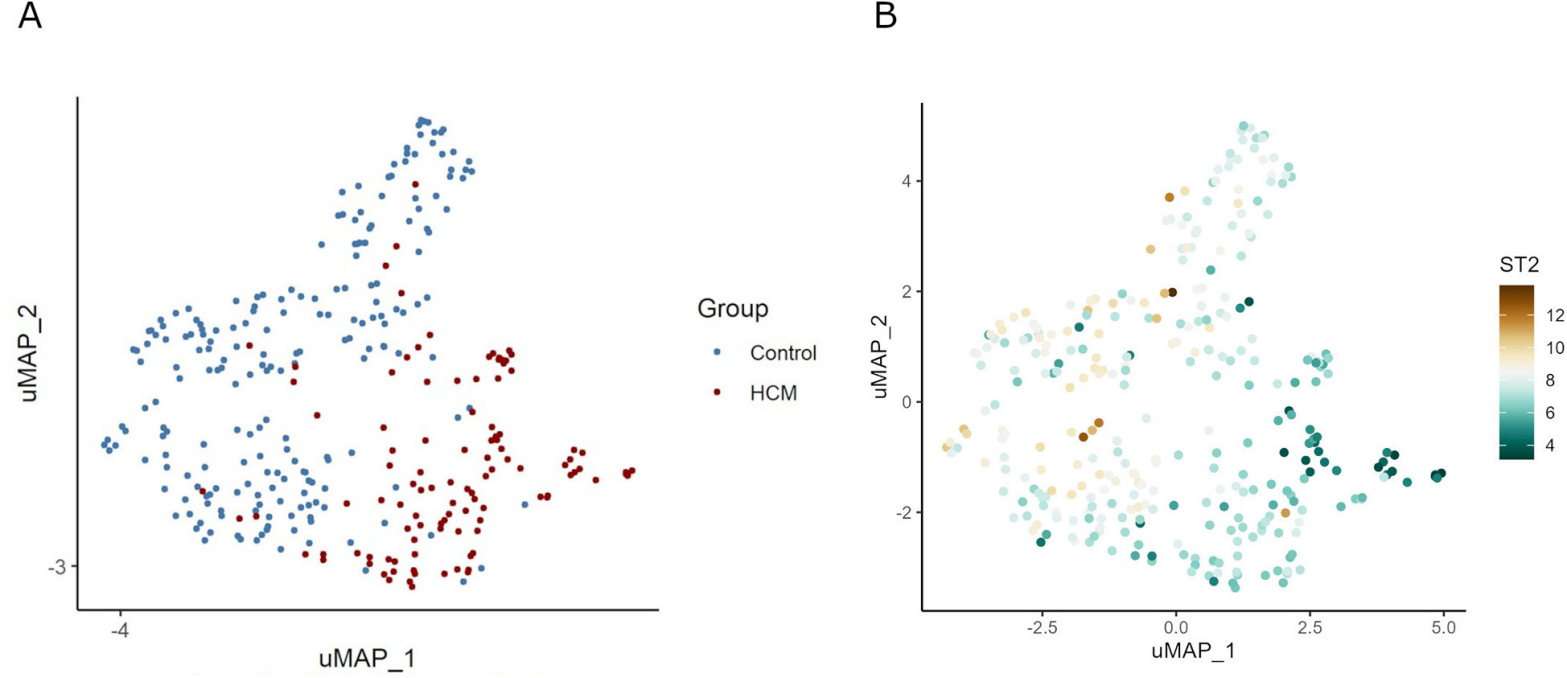
UMAP visualization of gene expression and *ST2* levels in HCM and control samples. Panel **(A)** demonstrates the clustering of samples into HCM and control groups, suggesting distinct variability in gene expression profiles between the two groups. Panel **(B)** shows the differential expression of the *ST2* gene across samples. In HCM samples, the expression level of *ST2* is significantly reduced.

**Figure 2:**
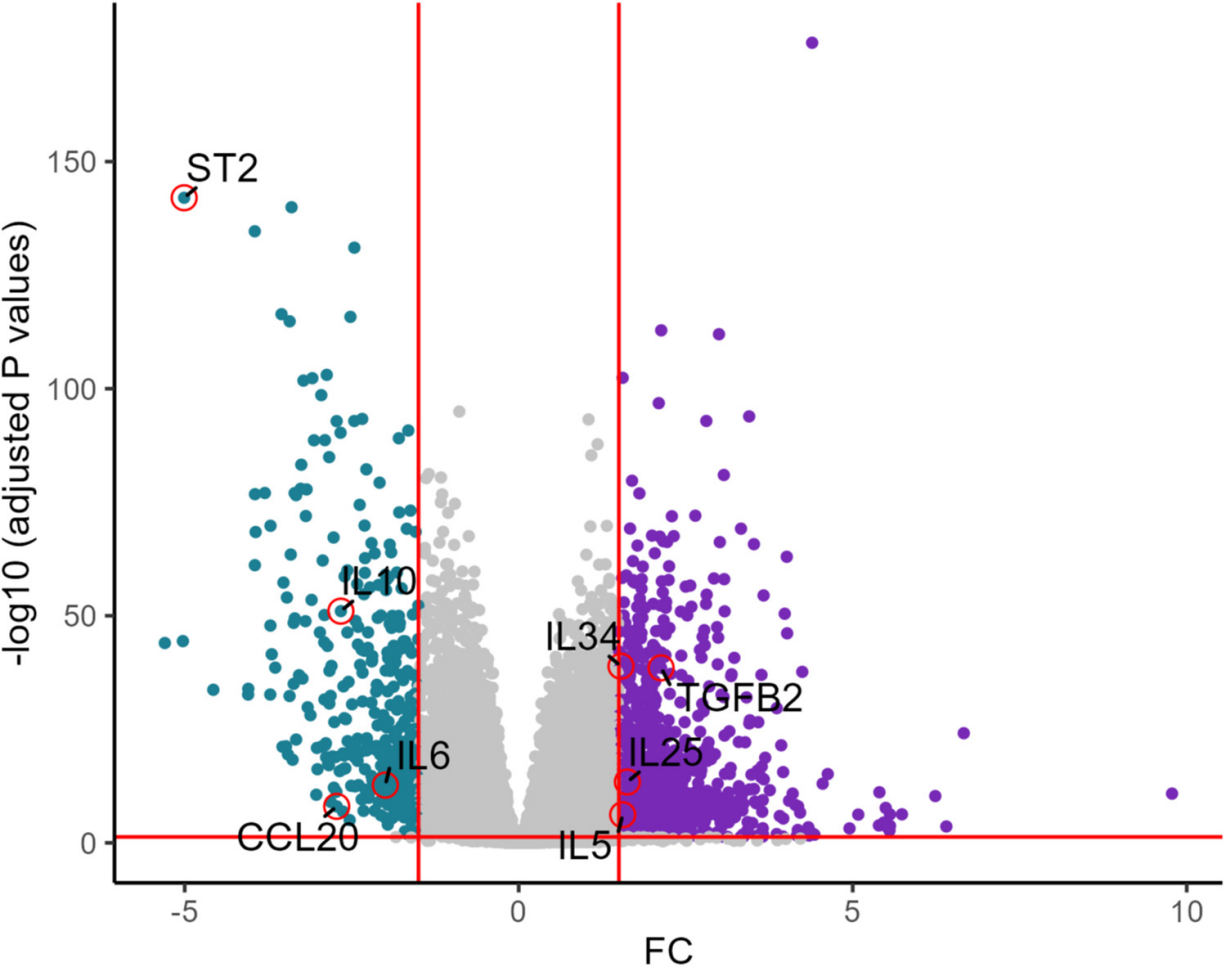
Volcano plot of key differentially expressed genes in HCM vs. healthy controls. The volcano plot shows differentially expressed genes between HCM and healthy controls. *ST2* is significantly downregulated along with *IL6, CCL20*, while genes such as *TGFB2, IL25, IL5*, and *IL34* are upregulated.

UMAP showed HCM cases and controls were separated well (Figure 1A). Based on the RNA-seq analysis of myocardial tissue samples, we identified a total of 2,660 upregulated and 403 downregulated genes when comparing HCM patients to healthy controls. Among these, the *ST2* gene was notably downregulated in HCM patients, with a significant overall log2 FC of −5.0 (*P* = 9.2 × 10^−^¹⁴³) (Figure 2). The UMAP analysis (Figure 1B) shows varying intensities of *ST2* expression across both clusters. This downregulation was consistently observed across multiple studies, as shown in Table 1, where the log2 FC for *ST2* ranged from −0.47 to −4.45. Additionally, the expression of fibrosis-related cytokines genes was analyzed, and several profibrotic cytokines genes, such as *TGFB2, IL5, IL25*, and *IL34*, were upregulated, while genes for *IL6*, and *CCL20* were downregulated. Conversely, the anti-fibrotic cytokine gene *IL10* was significantly downregulated (Table 2).

**Table 1:** Differential expression of the *ST2* gene in multiple HCM studies.

Author	Study	log2 FC	Adjusted *P* value
Liu, X	GSE130036	−2.08	1.68 × 10^−^^11^
Li, Y	GSE89714	−0.64	5.57 × 10^−^^1^
Margulies, KB	GSE141910	−4.12	3.17 × 10^−^^52^
Maron, B	GSE160997	−3.10	2.85 × 10^−^^5^
Ranjbarvaziri, S	GSE180313	−4.45	2.92 × 10^−^^15^
Wang, L	GSE133054	−0.47	5.78 × 10^−^^1^

The differential expression results of the *ST2* gene across various studies involving patients with HCM and healthy controls.

**Table 2:** Differential expression of the cytokines genes in HCM samples.

Gene role	Profibrosis	Anti-fibrosis
Growth factors	*TGFB2*↑	
Cytokines	*IL5*↑	*IL10*↓
	*IL6*↓	
	*IL25*↑	
	*IL34*↑	
	*CCL20*↓	

Genes are grouped according to their profibrotic or antifibrotic roles, based on their expression patterns in HCM cardiac tissue compared to controls.

### Correlation analysis

**Figure 3:**
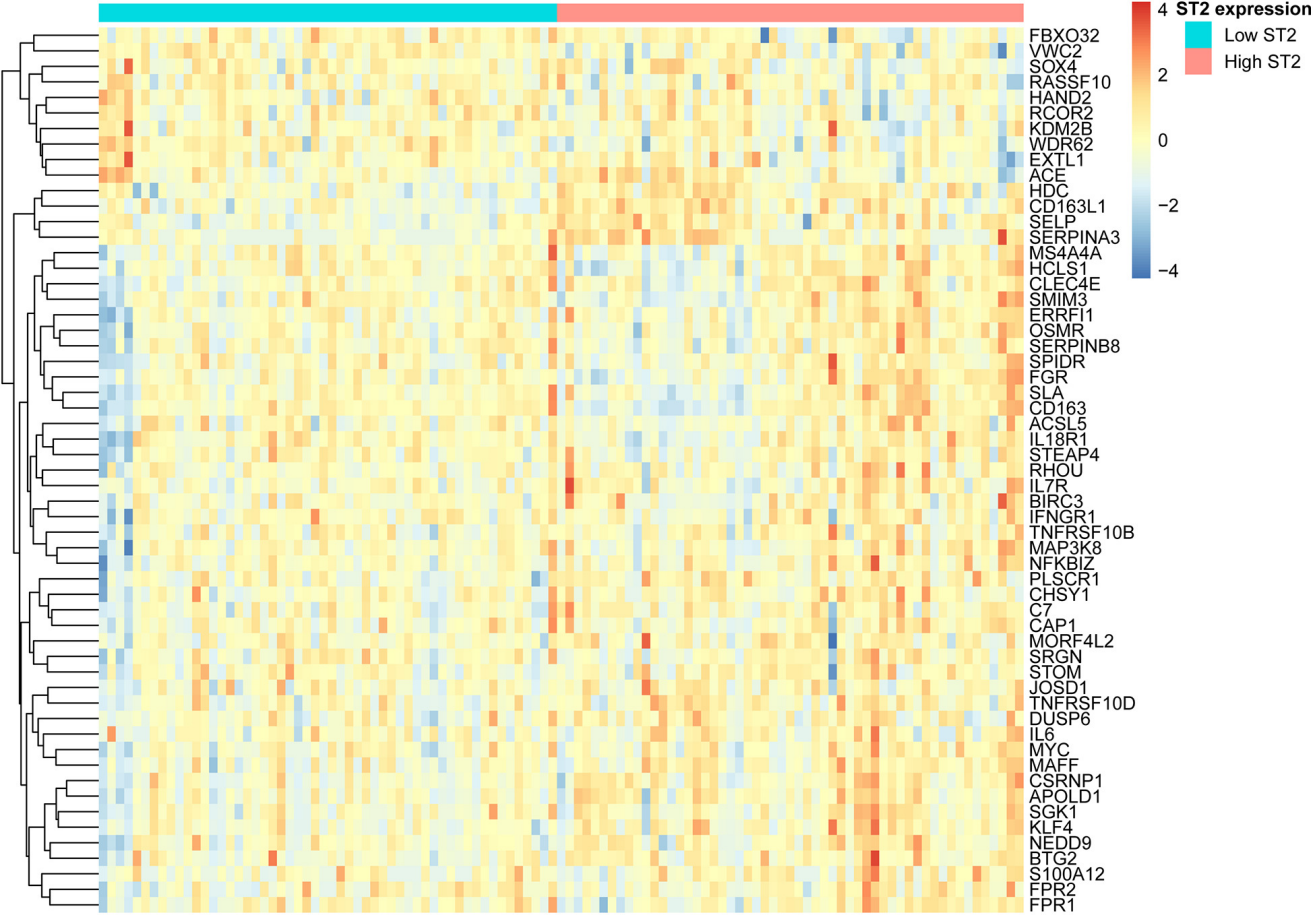
Heatmap of top *ST2* related genes categorized by *ST2* expression levels. This heatmap compares the expression levels of *ST2* significant correlated (*P* < 0.05, |r| > 0.5) genes across samples categorized by *ST2* expression levels. High and low *ST2* expression was defined by median.

Spearman's rank correlation coefficients were calculated to assess the relationship between *ST2* expression and genes involved in inflammation in HCM. The results revealed a significant positive correlation with *IL6* (*r* = 0.52, *P* = 8.6 × 10^−^^9^) and *CD163* (*r* = 0.62, *P* = 5.5 × 10^−^¹³). Additionally, the *MAP3K8* gene (*r* = 0.58, *P* = 6.8 × 10^−^^7^), which is integral to the MAP kinase pathways and cellular responses to stress and inflammation, also showed a correlation. Additionally, the *NFKBIZ* gene (*r* = 0.55, *P* = 8.3 × 10^−^^6^), a regulator of NF-κB involved in immune responses, was similarly correlated. Both NF-κB and MAP kinase are key signaling molecules in the IL-33/ST2 pathway, which has a corresponding trend with the downregulation of *ST2*. Furthermore, a notable negative interaction between pro-fibrotic and hypertrophic *ACE* (*r* = −0.55, *P* = 8.9 × 10^−^^9^) and the *ST2* was observed. The top 10 genes showing the most significant positive or negative correlations with *ST2* are listed in Table 3. Genes with |*r*| > 0.5 were shown in the heatmap (Figure 3).

**Table 3:** Genes most positively or negatively related to *ST2*.

*r*	Gene name	Adjusted *P*
Genes negatively correlated with *ST2*		
−0.61008	*SOX4*	3.78 × 10^−8^
−0.57980	*RASSF10*	7.85 × 10^−7^
−0.55307	*ACE*	8.93 × 10^−6^
−0.54553	*KDM2B*	1.70 × 10^−5^
−0.52846	*EXTL1*	6.98 × 10^−5^
−0.52659	*VWC2*	8.11 × 10^−5^
−0.51059	*RCOR2*	0.000281207
−0.50682	*HAND2*	0.000373665
−0.50379	*WDR62*	0.000468246
−0.50213	*FBXO32*	0.000468245
Genes positively correlated with *ST2*		
0.621505	*CD163*	1.10 × 10^−8^
0.585035	*ERRFI1*	4.75 × 10^−7^
0.581727	*SLA*	6.53 × 10^−7^
0.581324	*MAP3K8*	6.79 × 10^−7^
0.571079	*OSMR*	1.77 × 10^−6^
0.569567	*CLEC4E*	2.04 × 10^−6^
0.559082	*SERPINA3*	5.26 × 10^−6^
0.557568	*CSRNP1*	6.02 × 10^−6^
0.555344	*MYC*	7.32 × 10^−6^
0.553889	*NFKBIZ*	8.32 × 10^−6^

The genes most strongly negatively correlated with *ST2* include *SOX4, RASSF10, ACE, KDM2B,* and *EXTL1*, with correlation coefficients (*r*) ranging from −0.6101 to −0.5038. In contrast, the most strongly positively correlated genes include *ERRFI1, SLA, MAP3K8*, and *OSMR*, with *r* values ranging from 0.5509–0.6215.

### Enrichment analyses of DEGs

GSEA was performed to investigate the biological significance of *ST2*-related genes. Using both the GO and KEGG databases, the analysis revealed 1,080 upregulated and 198 downregulated GO terms, as well as 36 upregulated and no downregulated KEGG pathways. The top 20 positive and 20 negative terms in KEGG (Additional file 1: Supplementary Figure S1), each GO category—Biological Process (BP), Cellular Component (CC), and Molecular Function (MF)—were visualized using bubble diagrams.

### Downregulation of myocardial construction and structural adaptation in HCM

**Figure 4:**
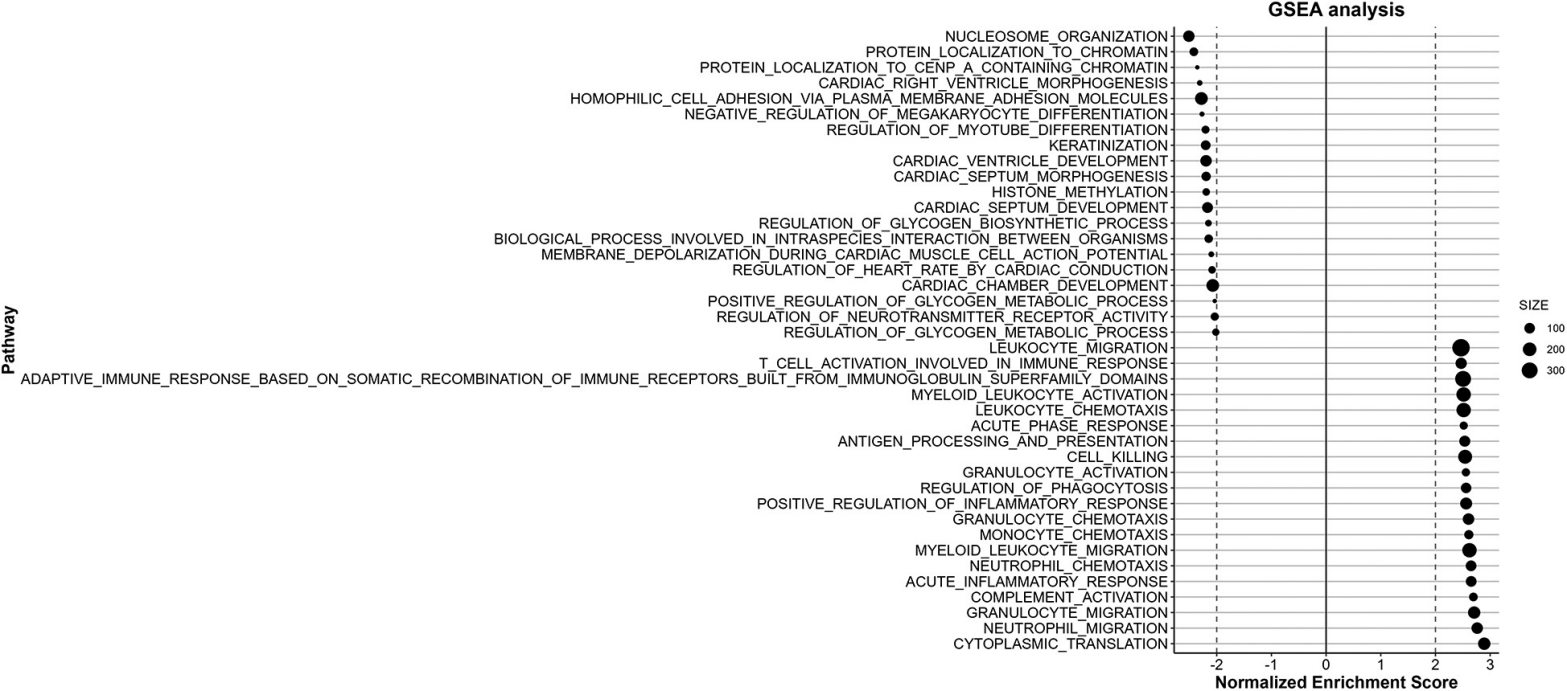
GSEA of BP correlated with *ST2* expression. The results of the BP analysis reveal the top 20 positively and negatively enriched pathways respectively. The downregulated pathways are primarily related to cardiac morphogenesis, while the pathways showing upregulation are mainly involved in inflammation and immune functions signaling processes.

The BP items highlighted that correlated genes were predominantly involved in immune response, inflammation, and cardiac morphogenesis. Processes related to myotube differentiation, cardiac septum development and morphogenesis, as well as cardiac ventricle development and formation were prominently featured and downregulated, indicating a robust involvement of these processes in HCM (Figure 4). The CC terms indicated a strong involvement in cellular structures related to immune function and protein synthesis. Key components such as signal transduction and cellular organization, as well as myosin filament, I band, myosin complex, actomyosin, and various types of junctions like tight junctions and adherents' junctions were highlighted. MF results emphasized histone modification activities, such as demethylase and methyltransferase activities, underscoring their role in gene expression alterations during HCM (Additional file 2: Supplementary Figure S2).

### Altered inflammation and fibrosis pathways in HCM

**Figure 5:**
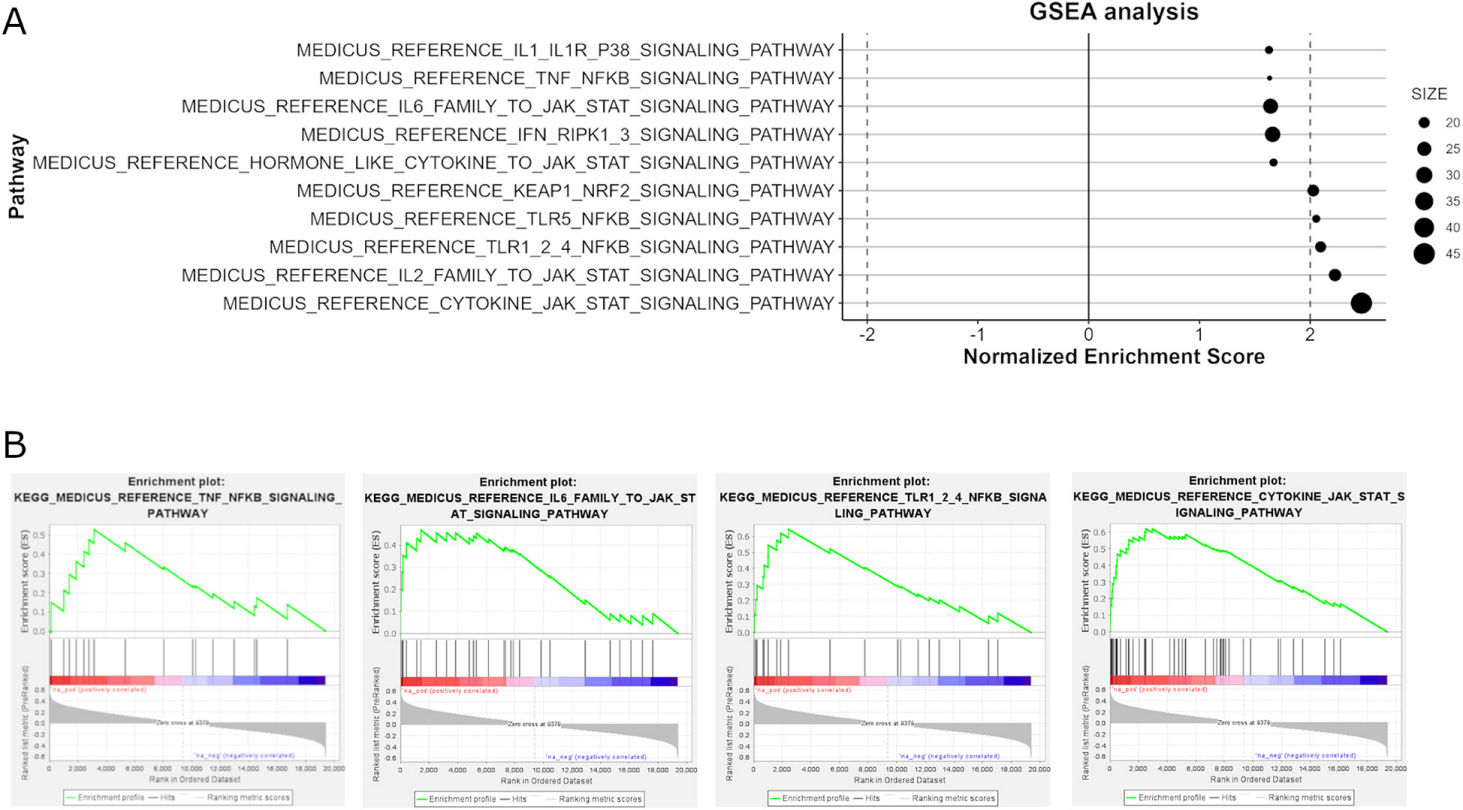
The analysis of KEGG pathways involved in immune response signaling through GSEA. Panel **(A)** identifies key upregulated pathways associated with inflammatory responses, such as IL-2, IL6, cytokine, JAK-STAT signaling, TLR (Toll-like Receptor) pathways, P38 signaling, and TLR5/TNF-NF-κB signaling, which are significantly enriched. Panel **(B)** provides enrichment plots for selected pathways.

Enrichment analysis revealed significant alterations in pathways related to immune system activity. Specifically, the most upregulated pathways in BP enrichment were mainly concentrated in inflammation and immune activation, such as neutrophil activation, T cell activation, adaptive immune reaction, response to type II interferon, leukocyte and monocyte chemotaxis, and cytokines production were notably upregulated. The KEGG pathway analysis also highlights significant dysregulation in several pathways associated with HCM, particularly those involved in inflammatory responses and cellular stress mechanisms. Pro- inflammatory pathways such as JAK-STAT signaling, TLR-NF-κB signaling, P38 signaling, TNF-NF-κB are prominently significantly enriched (Figure 5). Additionally, pathways related to oxidative stress response (KEAP1/NRF2 signaling) and translation initiation are also enriched. What's more, corresponding with *ACE* gene upregulation, the RAS pathway is upregulated (normalized enrichment score = 1.5, *P* = 0.04, FDR = 0.17).

### CIBERSORTx results of *ST2* and immune cells

**Figure 6:**
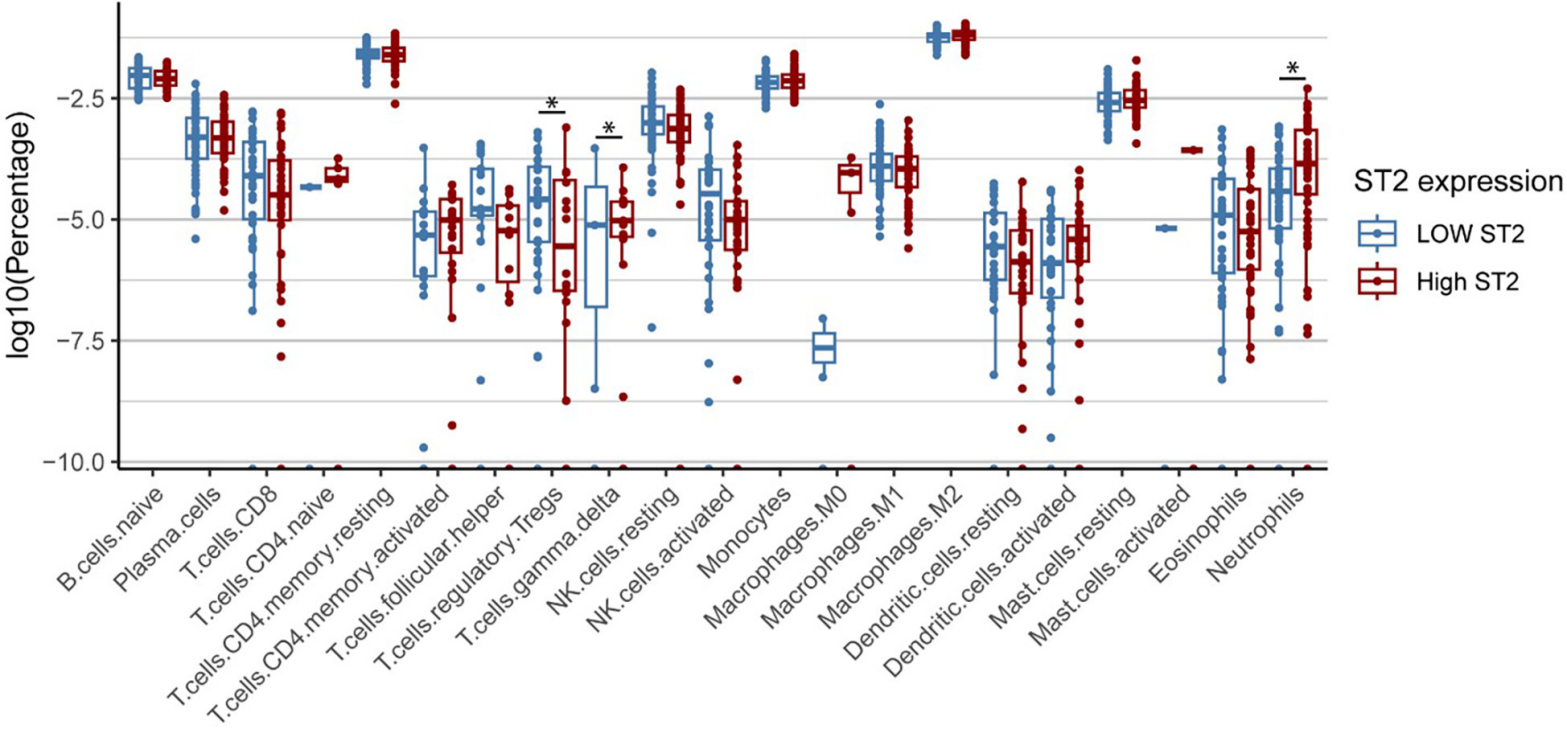
Differential *ST2* expression across immune cell types HCM. Cell type composition differences between high and low *ST2* expression groups in HCM patients. Box plots show significantly higher percentages of regulatory T cells (Tregs) in the low *ST2* group compared to the high *ST2* group, while higher percentages of neutrophils and γδ T cells are observed in the high *ST2* group. High and low *ST2* expression were defined by the median normalized *ST2* value. Statistical significance was assessed using the Wilcoxon test.

*ST2* gene correlation analysis with cell types derived from CIBERSORTx in HCM samples revealed a significant negative correlation with the proportion of regulatory T cells (Tregs), with a correlation coefficient (r) of −0.35 (*P* = 0.0002), suggesting that lower *ST2* expression is associated with increased Treg infiltration. Conversely, a strong positive correlation was found between *ST2* expression and the proportions of neutrophil (*r* = 0.39, *P* = 2.7 × 10^−^^5^) and gamma delta T cells proportions (*r* = 0.26, *P* = 0.0055), indicating that reduced *ST2* levels coincide with a lower presence of neutrophils (Figure 6).

### PPI network analysis and functional modules’ construction of *ST2* gene

**Figure 7:**
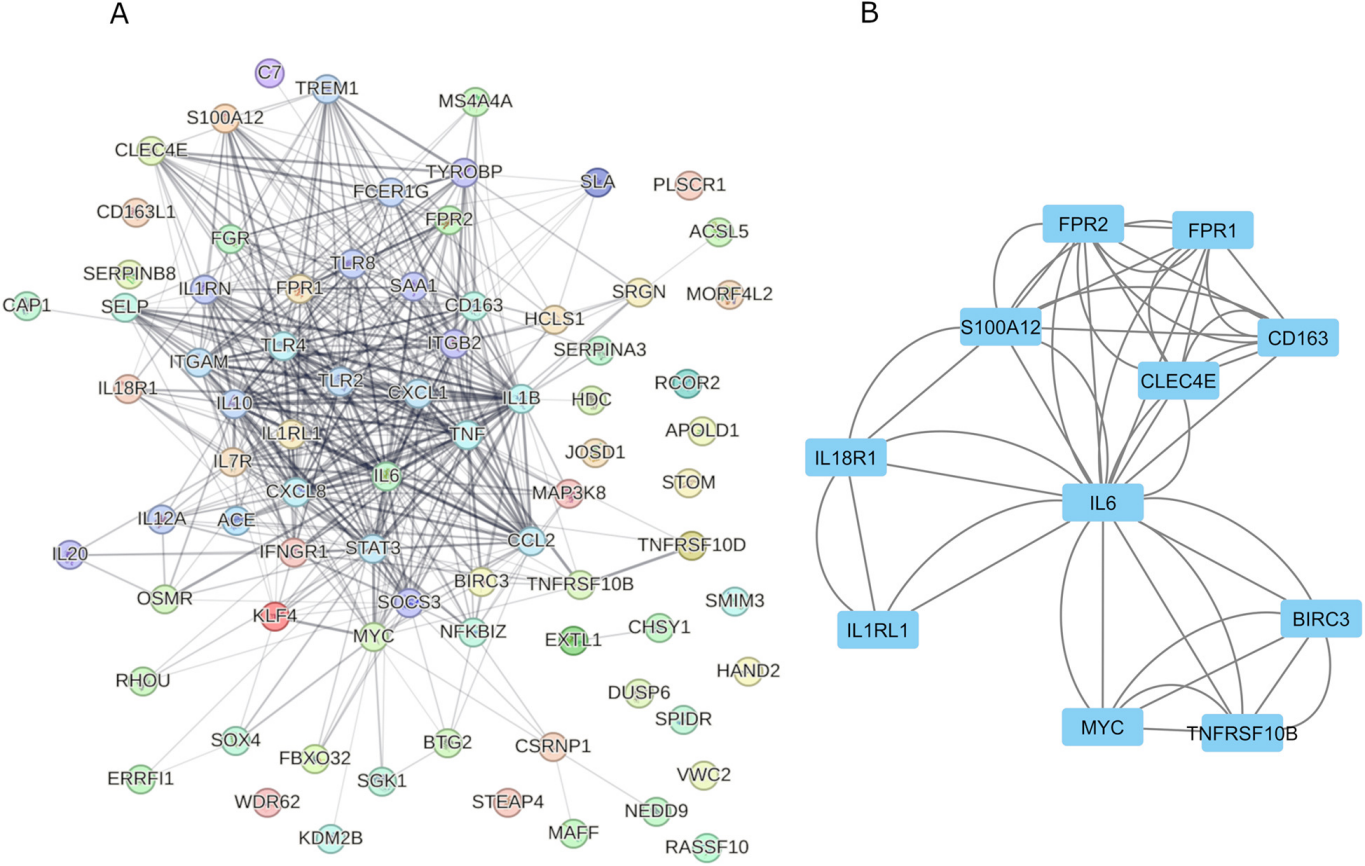
PPI networks and key modules constructed using STRING and cytoscape. Panel **(A)**: Displays a comprehensive PPI network involving *ST2(IL1RL1)* related genes, comprising 472 edges and 78 nodes. Within this network, the *ST2 (IL1RL1)* gene is highlighted as a pivotal component due to its significant interactions with other inflammatory mediators. Panel **(B)**: Utilizes MCODE plugin within CytoHubba to conduct a focused analysis, emphasizing *IL6* as a central node among the top 11 nodes and underscoring the centrality of the *ST2 (IL1RL1)* gene in the identified key modules.

**Figure 8:**
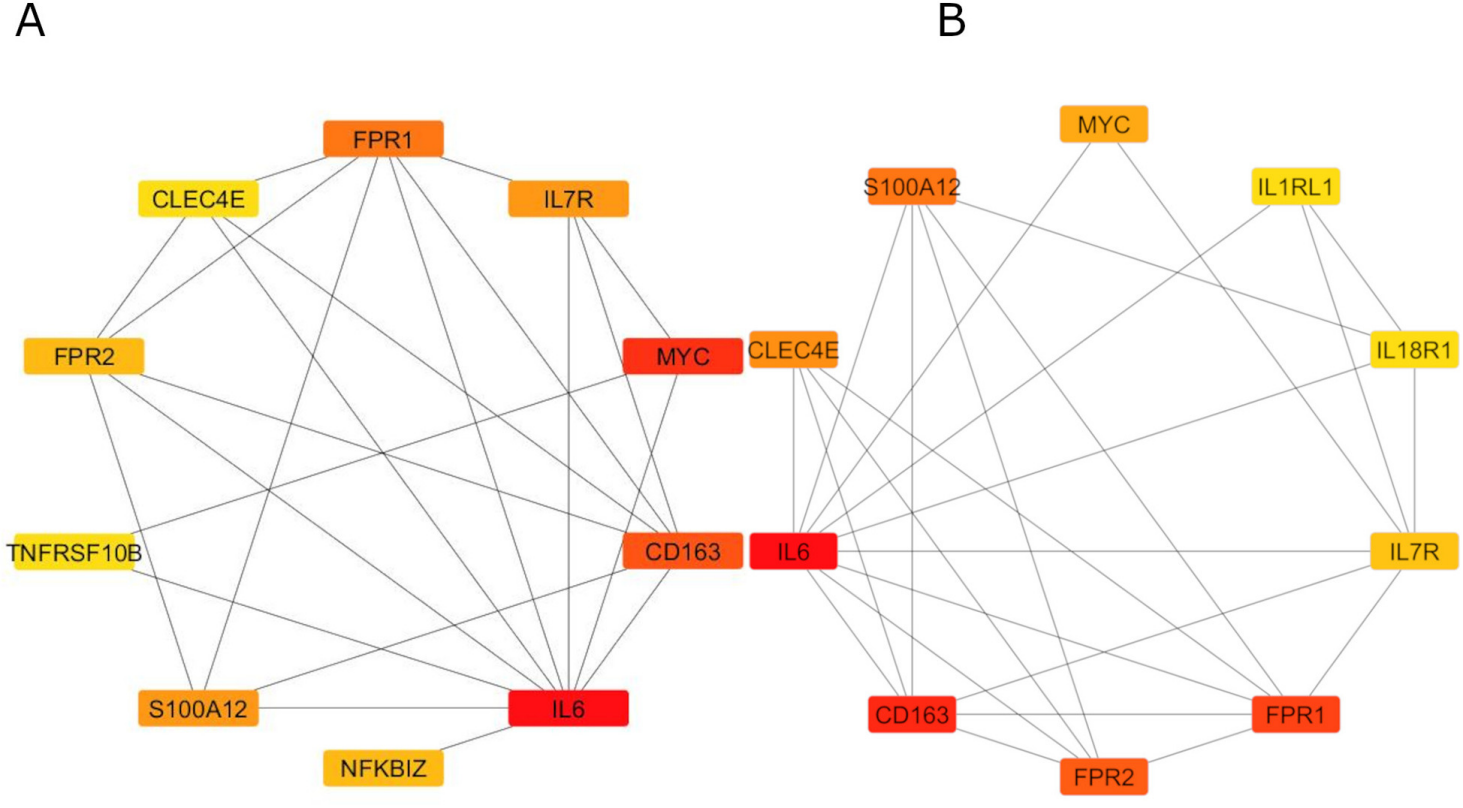
Degree centrality and MCC algorithms of CytoHubba analysis. Panel **(A)** reveals Degree Centrality scores, while Panel **(B)** shows MCC results, both identifying *IL6* and *CD163* as the highest-ranking hub genes.

A PPI network of *ST2* and its related genes was constructed using the STRING database (Figure 7A) and subsequently analyzed with Cytoscape. The molecular complex detection (MCODE) plugin identified a significant module consisting of 11 nodes, highlighting *IL6* as a central node with direct connections to several key inflammatory genes, including *IL7R, CD163, FPR1, FPR2*, and *S100A12* (Figure 7B). CytoHubba analysis using degree centrality and Maximal Clique Centrality (MCC) algorithms identified *IL6, CD163, FPR1, FPR2, S100A12, MYC, TNFRSF10B, IL1RL1 (ST2), IL18R1*, and *CLEC4E* as the top hub genes within the network (Figures 8). Specifically, *ST2* showed significant interactions, particularly with *IL18R1, IL7R,* and *TNFRSF10B*, suggesting its involvement in cytokine signaling pathways. Additionally, *MYC* was found to be closely connected with *IL6* and other hub genes, underscoring its role in the inflammatory network. STRING analysis confirmed these findings, depicting a complex network where *IL6* maintains extensive connectivity with multiple genes, including *NFKBIZ* and *ST2*, further reinforcing its central role in the inflammatory response.

## Discussion

The etiology of HCM is complex, with sarcomere gene mutations being the most widely recognized cause due to the resulting myocardial cell abnormalities. However, these mutations do not fully explain the significant myocardial fibrosis and malignant arrhythmias commonly observed in HCM. ST2 is a clinically monitorable biomarker, yet its role and impact in HCM remain underexplored. This article presents the first observation of significant downregulation of the *ST2* gene in a large HCM cohort, revealing its potential involvement in modulating inflammatory and fibrotic pathways. These findings offer a plausible mechanism that may explain the fibrosis observed in HCM.

### Impact of the IL-33/ST2 pathway on the HCM

The IL-33/ST2 pathway is involved in many cardiovascular diseases, including ischemic and valvular heart disease, myocardial infarction, heart failure, myocarditis, and cardiomyopathies. The IL-33/ST2 pathway is a vital mediator of cardiac protection, however it also can exacerbate cardiac remodeling in some situation like post-myocardial infarction (24). There may be a different balance in different disease situations, and this is the first time that we investigate the effect of IL-33/ST2 pathway in HCM.

In general, IL-33 binds to the ST2l and interleukin-1 receptor accessory protein receptor complex, activating myeloid differentiation primary response protein 88 (25), which recruits Interleukin-1 Receptor-Associated Kinase (IRAK) 4 to activate IRAK1. IRAK1 then interacts with TNF Receptor-Associated Factor 6, activating Transforming Growth Factor Beta-Activated Kinase 1(TAK1) (26). Subsequently, TAK1 triggers two pathways: the IκB Kinase pathway, activating NF-κB and calcium mobilization, and the MAPK pathway leading to cytokine production like TNF-α, IL-1, and IL-6 (27). These soluble molecules are released in the extracellular microenvironment and develop a suitable immune response to shut down fibrosis, inflammation, and ventricular remodeling to safeguard the heart (28). In our research, we observed the extreme downregulation of *ST2* gene with a log2 FC of −5.0, and corresponding downregulation of downstream in MAPK and NF-κB related genes, as well as the *IL6*, in HCM patients. This pattern suggests that the protective effects of the IL-33/ST2 pathway may be inhibited in HCM.

Sarcomere gene mutations are more commonly identified in children and adolescents. Interestingly, a similar animal research showed with age, *ST2* expression decreased followed by a significant increase in periarteriolar fibrosis in transverse aortic constriction model (29). Another animal study revealed that the lack of *ST2* led to a more pronounced systolic dysfunction, left ventricular hypertrophy, adverse remodeling, myocardial fibrosis, and worse survival in both ischemic and nonischemic heart failure (30). These experimental findings suggest that the downregulation of *ST2* and the dysregulation of the IL-33/ST2 axis may also contribute to the pathophysiology of HCM, explaining a possible pathogenesis older individual with HCM.

### Imbalance in inflammatory and fibrotic cytokines and pathways in HCM

The inflammatory cytokines/chemokines released following the IL-33/ST2 interaction depends on the types of immune cells that express *ST2* (28). Based on our differential expression analysis results, there is an imbalance in the expression of main pro-inflammatory and anti-inflammatory cytokines in HCM. Generally, IL-10 is a well-known anti-inflammatory property (31), and its reduced expression indicates a compromised anti-inflammatory mechanism. IL-6 can act as either anti-inflammatory or pro-inflammatory cytokines depending on the context (32), its production for a short time after heart injury can help heal and protect the heart. However, if IL-6 is produced for too long or in excess, it can lead to heart enlargement and heart failure. The protective effects of IL-6 come from its normal signaling on cell membranes, while its harmful, pro-inflammatory effects happen through a different process called trans-signaling (33). IL-25, IL-34 are pro-fibrosis factors (34). TGF-β2 plays distinct role in the fibrotic process in organs. The upregulation of TGF-β2 has been linked to increased inflammatory cell infiltration and exacerbated fibrosis (35, 36), as observed in conditions such as myocardial infarction and ischemic cardiomyopathy (37). IL-5 and IL-25 are key cytokines associated with the IL-33/ST2 pathway in Th2-type immune responses (38). In progenitor innate lymphoid cells (ILCs), TGF-β2 can promote the expression of ST2, essential for the functioning and maintenance of ILC2s, which produce cytokines like IL-5. IL-25 triggers ILC2s to produce IL-5 and works synergistically with IL-33 to enhance ILC2 function (39). In our HCM samples, the observed increase expression in *TGFB2* coupled with a decrease in *ST2* expression, suggests a complex regulatory mechanism where higher *TGFB2* levels do not lead to increased *ST2* expression in the cardiac environment. This discrepancy might indicate disrupted signaling pathways or feedback mechanisms specific to the heart, potentially leading to fibrosis due to similar inflammatory pathways.

The enrichment analysis identified a marked imbalance between pro-inflammatory and anti-inflammatory pathways in HCM. Specifically, we observed significant upregulation of pathways involved in inflammation and immune activation, such as neutrophil activation, T cell activation, cytokines production and chemotaxis, as well as adaptive immune responses. The upregulation of these pathways, including the JAK-STAT signaling, TLR-NF-κB, and p38 MAPK pathways, TNF-NF-κB even RAS signaling pathway underscores the heightened inflammatory state in HCM. These pro-inflammatory pathways are known to contribute to myocardial fibrosis and hypertrophy by promoting the production of cytokines and chemokines that drive the recruitment and activation of immune cells in the cardiac tissue. However, upregulation of anti-inflammation or fibrotic pathways can only be found in KEAP1/NRF2 signaling.

The balance between these cytokines and pathways reflects the dual roles of immune responses in HCM, where both inflammation and anti-inflammation mechanisms are at play, significantly impacting fibrosis progression and cardiac tissue remodeling. However, overall, the imbalance between anti-inflammatory and pro-inflammatory factors, with a significant upregulation of pro-inflammatory and pro-fibrotic factors, accompanying by downregulation of anti-inflammatory factors may explain the widespread fibrosis and remodeling seen in HCM.

### Immune cells infiltration in HCM

Immune cells play significant roles in various types of heart disease, including HCM. Regulatory T cells (Tregs) play a crucial role in modulating immune responses and maintaining immune tolerance. Typically, Tregs suppress excessive immune responses and protect tissues, as well as in overload conditions, such as in HCM (40). Previous studies have shown that the number of Tregs decreases, leading to a loss of their protective effects in HCM (41). In the early stages of injury, Tregs can inhibit inflammatory responses, however, during heart failure, Tregs can alter their phenotype and function, potentially exacerbating the condition (42). *ST2* is expressed as a marker on a subset of Tregs, which are believed to help mitigate fibrosis and promote healing processes. Through IL-33/ST2 signaling, Tregs can exert powerful cytokines like IL-10 and TGF-β, aiding in the resolution of inflammation and facilitating tissue repair (43, 44). In our immune infiltration analysis, we found that samples with low *ST2* expression had a higher proportion of regulatory Tregs, accompanied by decreased expression of *IL10*, that implies although the proportion of Tregs is higher, their functionality may be impaired (37).These changes in cytokine expression, combined with the increased proportion of Tregs with impaired function, we speculate that while the body may be attempting to control inflammation by increasing Treg numbers, the lack of *ST2* prevents these Tregs from effectively performing their anti-inflammatory roles (45). This results in a sustained inflammatory state and tissue damage. There is limited research about neutrophils and gamma delta T cells in HCM. Although the exact results of *ST2* downregulation and increasing neutrophils and gamma delta T cells remains unclear, these findings suggest that *ST2* downregulation may disrupt the balance of immune cell populations, potentially exacerbating the inflammatory and pathological processes in HCM.

### Central role of *IL6* and *ST2* in immune response pathways in HCM

Our study reveals that the downregulation of *ST2* in HCM is intricately linked to a dysregulated inflammatory gene network, with *IL6* emerging as a central node. Although extensive research has focused on IL-6 in cardiovascular diseases, heart failure, and valvular disease, its role in HCM remains largely unexplored. The strong association between IL-6 and other inflammatory mediators suggests that the dysregulation of this cytokine could contribute significantly to the pathophysiology of HCM. Moreover, IL-6 plays a central role in myocardial fibrosis that depends on the activation of the MAPK and STAT3 pathways (46), experiments have shown that this upregulation contributes to cardiac hypertrophy and early mortality in mice (47), suggesting that of IL-6/STAT3 signaling pathway may promote cardiac fibrosis and hypertrophy, and offer a new target for cardiomyopathy (48). Our findings indicate that the IL6-JAK-STAT and NF-κB pathways are upregulated in HCM samples, similar to the research of Yao et al's, providing a possible impact of IL-6 and associated pathway in HCM (49).

Furthermore, the *MYC* oncogene is known to interact with IL-6 pathways, potentially enhancing *IL6* expression and thereby perpetuating this cycle of inflammation and fibrosis (50). Additionally, *ST2*'s interactions with *IL18R1, IL7R*, and *TNFRSF10B* suggest that reduced ST2 levels may disrupt protective cytokine signaling, exacerbating inflammation, and contributing to HCM pathogenesis.

### Breakdown of anti-inflammatory responses in HCM: the ST2, Treg, and IL-6 connection

Our analysis highlights a complex interplay with ST2, Tregs, and IL-6 as key players. Reduced IL-33/ST2 signaling could lead to IL-6 downregulation, which in turn triggers immune dysregulation (51, 52). While IL-6 typically supports the stability and function of Tregs, reduction in IL-6 disrupts Treg functionality, impairing their capacity to maintain immune tolerance through the STAT3 pathway (53, 54). Additionally, lower ST2 expression compounds this issue by further reducing IL-33/ST2-mediated activation of Tregs and their production of IL-10, crucial for their function. Furthermore, IL-6 downregulation affects M2 macrophage differentiation, marked by CD163 downregulation, which is consistent with our study (55). This leads to decreased IL-10 secretion, thereby weakening the immune tolerance mechanism. The reduced IL-10 availability further inhibits Treg functionality, perpetuating a pro-inflammatory state (56). Although Tregs may increase in proportion, their functional impairment allows persistent inflammation and fibrosis progression.

Overall, ST2, IL-6, and Tregs appear to be closely linked, and their collective dysfunction may potentially contribute to the breakdown of the anti-inflammatory system, thereby promoting ongoing inflammation and fibrosis in HCM. Given the nascent state of research on the IL-33/ST2 axis in HCM, further in-depth studies are crucial to explore these preliminary findings.

## Limitations

This study has several limitations. First, although we conducted bioinformatic analyses using data from 10 different databases, our findings are limited by the absence of experimental validation. Future studies should include both *in vitro* and *in vivo* experiments, along with plasma ST2 measurements, to confirm the functional significance of *ST2* downregulation and its connection to inflammatory pathways. Moreover, the immune infiltration analysis was based solely on transcriptomic data, and further studies are necessary to elucidate the underlying mechanisms more clearly; Finally, while our findings highlight key molecular pathways, further mechanistic studies are required to establish causal links between *ST2* downregulation and the progression of fibrosis and hypertrophy in HCM.

## Conclusion

Our study suggests that downregulation of *ST2* in HCM may disrupt the IL-33/ST2 signaling pathway, potentially contributing to impaired immune regulation and increased inflammation. This disruption is associated with elevated activity in pro-inflammatory pathways, such as JAK-STAT, NF-κB, and MAPK, alongside alterations in IL-6 and TGF-β2, which may contribute to myocardial fibrosis and hypertrophy in HCM. Additionally, the diminished protective role of *ST2* might be linked to the exacerbation of chronic inflammation and adverse cardiac remodeling in HCM. Further exploration of these pathways could offer new therapeutic strategies for managing fibrosis and the progression of HCM.

## Data Availability

The datasets presented in this study can be found in online repositories. The names of the repository and accession number(s) can be found below: Gene Expression Omnibus database (accession number: GSE133054, GSE130036, GSE141910, GSE106997, GSE89714, GSE145154, GSE160997, GSE180313, GSE188238, and GSE206978).

## Supplementary material

Supplementary Figure S1 **KEGG Analysis of Genes Correlated with ST2 Expression in HCM.** Top 20 positively and negatively enriched pathways in GESA analysis based in KEGG database. Significantly enriched pathways include inflammatory processes are predominantly upregulated.

Supplementary Figure S2 **GSEA of Top CC and MF Correlated with ST2 in HCM.** Panel A illustrates the GSEA of CCs, highlighting key structures involved in cellular organization and immune function. Panel B presents the GSEA results for MFs, emphasizing the critical role of histone modification activities in regulating gene expression. CC, cellular component; MF, molecular function.
