# Supplementary figures and images for: Interplay of ST2 downregulation and inflammatory dysregulation in hypertrophic cardiomyopathy pathogenesis
Source: Front Cardiovasc Med. 2025 Jun 4;12:1511415. doi: 10.3389/fcvm.2025.1511415 (PMC12174446; doi:10.3389/fcvm.2025.1511415)

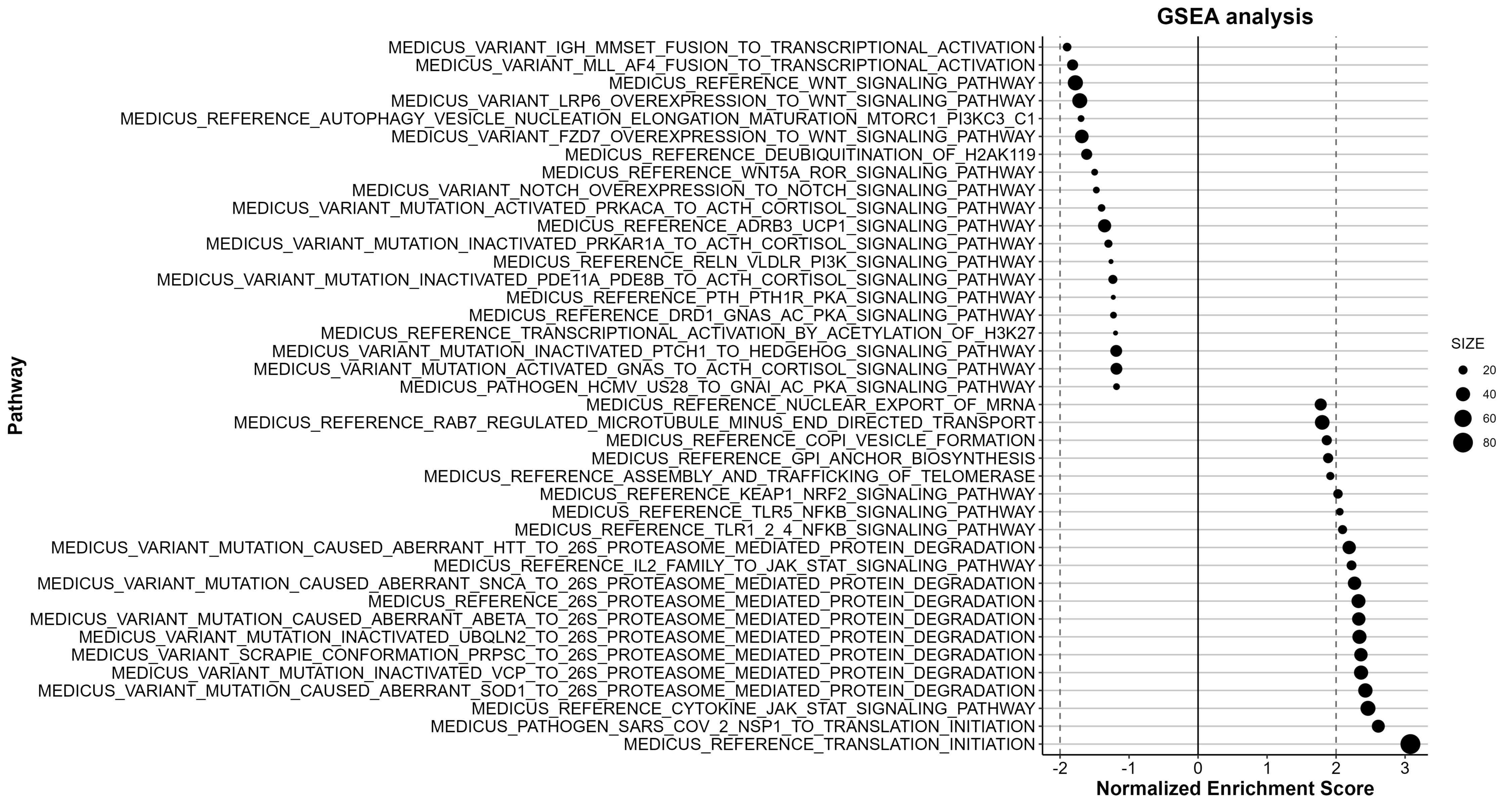

Supplement: Supplementary Figure S1 — KEGG Analysis of Genes Correlated with ST2 Expression in HCM. Top 20 positively and negatively enriched pathways in GESA analysis based in KEGG database. Significantly enriched pathways include inflammatory processes are predominantly upregulated. [file Image1.tif]

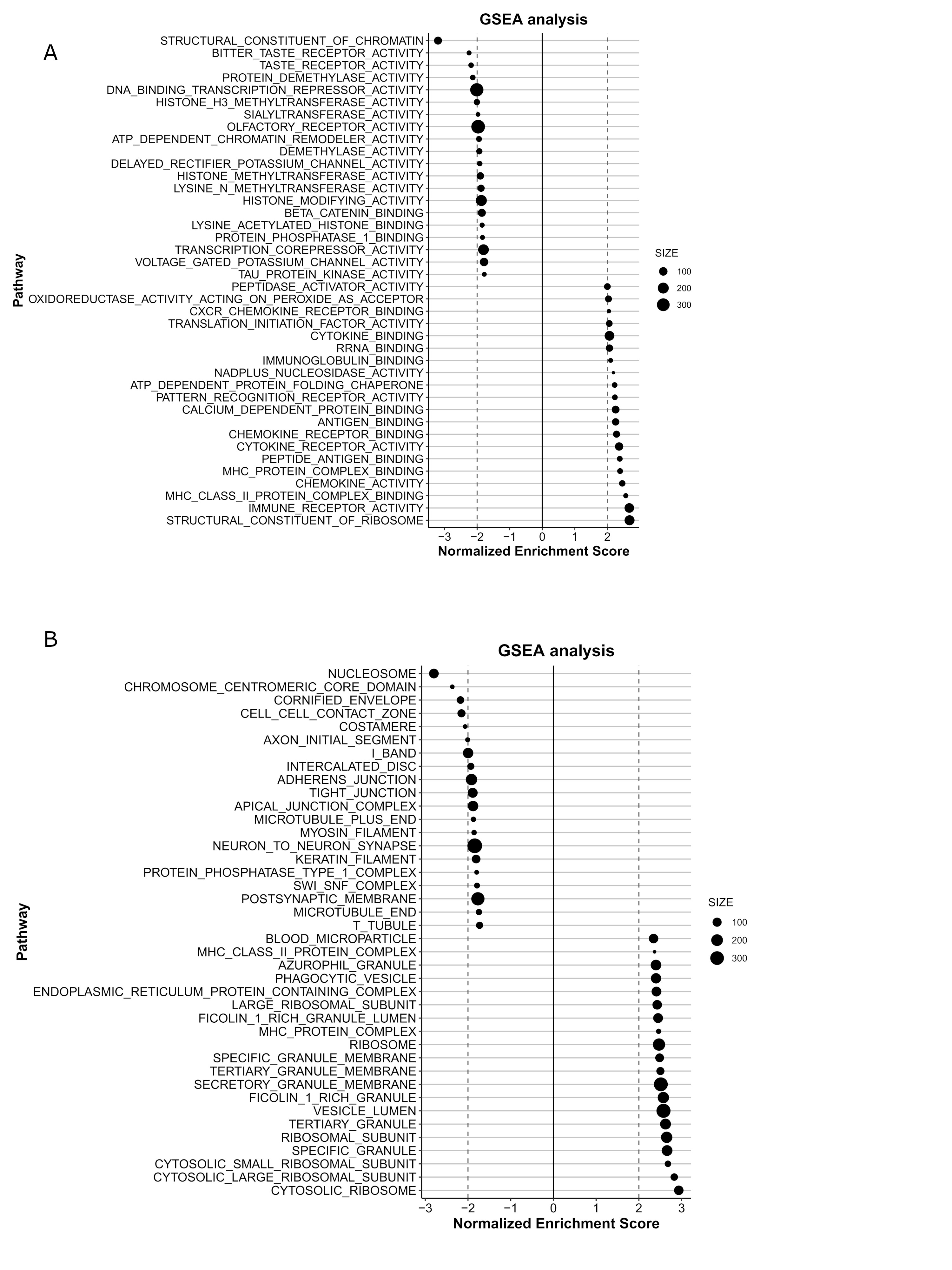

Supplement: Supplementary Figure S2 — GSEA of Top CC and MF Correlated with ST2 in HCM. Panel A illustrates the GSEA of CCs, highlighting key structures involved in cellular organization and immune function. Panel B presents the GSEA results for MFs, emphasizing the critical role of histone modification activities in regulating gene expression. CC, cellular component; MF, molecular function. [file Image2.jpeg]
